# HDAC6 regulates primordial follicle activation through mTOR signaling pathway

**DOI:** 10.1038/s41419-021-03842-1

**Published:** 2021-05-29

**Authors:** Tuo Zhang, Meina He, Lihua Zhao, Shaogang Qin, Zijian Zhu, Xinhua Du, Bo Zhou, Yi Yang, Xinfeng Liu, Guoliang Xia, Tengxiang Chen, Yuanxi Wang, Hua Zhang, Chao Wang

**Affiliations:** 1 Key Laboratory of Ministry of Education for Conservation and Utilization of Special Biological Resources in the Western China, College of Life Science, Ningxia University, 750021 Yinchuan, China; 2 Guizhou Provincial Key Laboratory of Pathogenesis & Drug Research on Common Chronic Diseases, Department of Physiology, School of Basic Medical Sciences, Guizhou Medical University, 550009 Guiyang, Guizhou China; 3 State Key Laboratory of Agrobiotechnology, College of Biological Sciences, China Agricultural University, 100193 Beijing, China; 4 Department of Biology, School of Basic Medical Science, Guizhou Medical University, 550025 Guiyang, Guizhou China; 5 Department of Pathology and Hepatology, the 5th Medical Centre, Chinese People’s Liberation Army General Hospital, 100039 Beijing, China; 6 RDFZ Xishan School, 100193 Beijing, China

**Keywords:** TOR signalling, Cell proliferation, Endocrine reproductive disorders

## Abstract

Primordial follicle pool established perinatally is a non-renewable resource which determines the female fecundity in mammals. While the majority of primordial follicles in the primordial follicle pool maintain dormant state, only a few of them are activated into growing follicles in adults in each cycle. Excessive activation of the primordial follicles accelerates follicle pool consumption and leads to premature ovarian failure. Although previous studies including ours have emphasized the importance of keeping the balance between primordial follicle activation and dormancy via molecules within the primordial follicles, such as TGF-β, E-Cadherin, mTOR, and AKT through different mechanisms, the homeostasis regulatory mechanisms of primordial follicle activation remain unclear. Here, we reported that HDAC6 acts as a key negative regulator of mTOR in dormant primordial follicles. In the cytoplasm of both oocytes and granulosa cells of primordial follicles, HDAC6 expressed strong, however in those activated primordial follicles, its expression level is relatively weaker. Inhibition or knockdown of HDAC6 significantly promoted the activation of limited primordial follicles while the size of follicle pool was not affected profoundly in vitro. Importantly, the expression level of mTOR in the follicle and the activity of PI3K in the oocyte of the follicle were simultaneously up-regulated after inhibiting of HDAC6. The up-regulated mTOR leads to not only the growth and differentiation of primordial follicles granulosa cells (pfGCs) into granulosa cells (GCs), but the increased secretion of KITL in these somatic cells. As a result, inhibition of HDAC6 awaked the dormant primordial follicles of mice in vitro. In conclusion, HDAC6 may play an indispensable role in balancing the maintenance and activation of primordial follicles through mTOR signaling in mice. These findings shed new lights on uncovering the epigenetic factors involved physiology of sustaining female reproduction.

## Introduction

Ovarian follicles are the basic and non-renewable reproductive unit of female mammals^1,2^. In mammalian ovaries, the majority of follicles reserved are primordial follicles, which are maintained at dormant or quiescent state for as long as 50 years in humans or for more than 1 year in mice. Only a small portion of dormant primordial follicles are progressively recruited into the growing follicle pool each time in adults, which is called primordial follicle activation^3^. Obviously, the balance between quiescent and activated state of the primordial follicles is crucial for female fecundity. Excessive activation of primordial follicles will lead to exhaustion of the resting follicle reserve and result in premature ovarian failure (POF), which seriously affect female physical and mental health^4^. Unfortunately, there is hardly any effective measurements to help these patients so far because the acknowledgement of the mechanisms governing the activation and dormant of primordial follicles is still obscure.

The primordial follicle consists of a quiescent oocyte and surrounding flattened primordial follicle granulosa cells. Previous studies including ours have implied that oocyte derived molecules, such as phosphatidylinositol 3-kinase (PI3K), cell division cycle 42 (CDC42), E-cadherin/N-adherin (E-cad/N-cad), and newborn ovary homeobox (NOBOX)^5–11^, as well as oocyte and pfGCs expressed Sirtuin 1 (SIRT1) and transforming growth factor β (TGF-β)^12,13^, are pivotal for the sustainment of primordial follicles after birth. On the other hand, the activation of primordial follicle is a complex and coordinated process among individual oocyte, pfGCs, and even the surrounding micro-environment^14^. Actually, many molecules mentioned above are involved in the activation process of primordial follicle originated from the pfGCs, of which mTORC1 signaling in pfGCs and PI3K signaling in oocytes is the dominant pathway^15^.

Although the technique of in vitro activation (IVA) of primordial follicles based on these findings have achieved to help POF patients producing fertilizable oocytes for successful reproduction, the efficiency is quite low^16,17^. And it is hard to unveil the pathological mechanism of POF so far because there is very limited knowledge about how primordial follicle dormancy is finely tuned after birth.

Most recently, we have proved that SIRT1, one of the members of histone deacetylase family, contributes to sustain primordial follicles in mice, implying the importance of epigenetic modification effect in the process^12^. In line with this, an overexpression of histone deacetylase 6 (*Hdac6*) extends reproductive lifespan of mice since an increased folliculogenesis and particularly secondary and antral follicles are found in 19-month-old mice compared with 16-month wild-type mice^18^. HDAC6 belongs to class IIb of HDAC family, and is a microtubule-associated deacetylase^19^. Physiologically, it predominantly functions in the cytoplasm by deacetylating various substrates so as to regulate cell migration, cell cytoskeleton, cell motility, and cell immune synapse formation, and even misfolded proteins degradation^20–26^. HDAC6 contains two intact catalytic domains that located respectively at the N-terminal and the central regions of the protein, which is different from those of SIRT1. Additionally, it is not only important for regulating chromatin compaction and meiotic apparatus assembly in mouse oocytes, but plays essential part in *ARID1A*-mutated ovarian cancer^27–30^. Therefore, we wonder if HDAC6 was also involved in sustaining primordial follicles.

Briefly, we found that HDAC6 participates in sustaining primordial follicle quiescent in newborn mice. We proved that either inhibiting or knocking down HDAC6 promoted the activation of primordial follicles, while overexpression of *Hdac6* retarded the activation of primordial follicle. HDAC6 regulates primordial follicles quiescent through regulating the expression of mTOR signaling. Collectively, we hypothesize that HDAC6 plays an indispensable role in sustaining primordial follicles through affecting the expression of mTOR signaling pathway.

## Materials and methods

### Animal

Adult CD-1 mice were purchased from Beijing Vital River Laboratory Animal Technology Co., Ltd. and bred to male mice of the same strain. Mice were housed under controlled lighting (12 h light, 12 h dark) and temperature (24–26 °C) conditions with free access to food and water. Vaginal plug detection was considered as 0.5 day post coitus (dpc). The first 12 h after birth was considered as 0 day postpartum (dpp). All procedures were conducted in accordance with the guidelines of and approved by the Animal Research Committee of the China Agricultural University.

### Whole ovary and cortex fragments isolation

The newborn mice were sacrificed at the designated times. The ovaries of the newborn mice were micro-dissected in cold phosphate buffered saline (PBS) under microscope. Make sure that the structure of whole ovaries was not damaged during the separation procedure. When isolating the cortex fragments under a steroid microscope, a syringe of 1 ml with needle was applied to carefully separate the ovarian cortex from the medulla tissue.

### Ovary organ culture

The dissected ovary was cultured in six-well culture plates (Nest, Jiangsu, China) in 1200 μl of Dulbecco Modified Eagle Medium/Ham F12 nutrient mixture (DMEM/F12) (Gibco, Life Technologies, CA) with insulin-transferrin-sodium selenite (Sigma, 1:100, USA) and penicillin–streptomycin solution at 37 °C, 5% CO_2_ and saturated humidity. The female mice ovaries were randomly assigned. Same number of ovaries in each group. The culture medium was exchanged every other day. The final concentrations of Tubastatin A (TubA in short) (S8049; Selleck, Shanghai, China), Rapamycin (Rap in short) (S1039; Selleck, Shanghai, China), and ISCK03 (I6410; Sigma, USA) were 4 μM, 2.5 μM, and 5 μM, respectively. The control group was supplemented with the same volume of dimethyl sulfoxide (DMSO).

### Immunofluorescence and immunohistochemistry

Freshly isolated ovaries were fixed in cold 4% paraformaldehyde overnight, dehydrated in gradient alcohol, embedded in paraffin, and sectioned at 5 μm. Ovarian sections were deparaffinized, rehydrated and subjected to antigen retrieval with 0.01% sodium citrate buffer (pH 6.0) at high temperature (95–98 °C) for 16 min. The sections were then rinsed thoroughly with PBS and blocked with normal donkey serum in PBS for 1 h at room temperature and incubated with primary antibodies overnight at 4 °C. The primary antibodies used are listed in Table S1. Next, ovarian sections was rinsed thoroughly with PBS and incubated with Alexa Fluor 488- or 555- conjugated secondary antibody for 1 h at 37 °C. The ovarian sections were rinsed thoroughly with PBS, stained with DAPI for 5 min and sealed in anti-fade fluorescence mounting medium (C1210, Applygen, China) with coverslips. Immunohistochemistry was performed using Histostain™-SP Kits (PV-9001, ZSGB-BIO, China) and DAB peroxidase substrate kits (ZLI-9017, ZSGB-BIO, China) according to the manufacturer’s protocols. Sections were examined and photographed using Nikon 80i or Nikon A1 laser.

### Total follicle, primordial follicle, and primary follicle counting

The serial sections of each ovary (8 μm thick) were stained with hematoxylin. Only when follicles containing clearly visible nucleus of oocytes in each individual section of every ovary were counted. The serial sections of each ovary were counted before statistical analysis. Maximum and minimum were excluded. A double-blind experiment was performed during follicle number counting. The follicles were distinguished from each other as follows: primordial follicle (a single oocyte surrounded by several flattened pre-granulosa cells) and primary follicle (an enlarged oocyte surround by a mixture of squamous and cuboidal granulosa cells).

### Western blotting analysis

Each protein sample obtained from at least six ovaries were extracted in WIP Tissue and cell lysis solution containing 1 mM phenylmethylsulfonyl fluoride (8553 S, Cell Signaling Technologies, USA) according to the manufacturer’s instructions. The protein concentration was measured by a BCA assay (P0012, Beyotime, China). The samples were separated on 10% SDS-PAGE and then transferred to polyvinylidene fluoride membranes (IPVH00010, Millipore, USA). The membranes were blocked with 5% skim milk powder for 1 h at room temperature and then incubated with appropriate primary antibodies overnight at 4 °C. The primary antibodies used are listed in Table S1. The membranes were rinsed thoroughly with TBST. The membranes were incubated with the secondary antibody (1:5000, ZSGB-BIO, China) for 1 h at room temperature and rinsed thoroughly with TBST. The membranes were visualized using a SuperSignal West Pico Chemiluminescent Detection System (Prod 34080, Thermo, USA). β-actin (CW0096M, Cwbiotech, China) was used as the intrinsic control.

### Real-time PCR analysis

Total RNA obtained from at least six ovaries was extracted using TRIzol reagent (Invitrogen, Carlsbad, CA, USA) according to the manufacturer’s protocol. First-strand cDNA was synthesized by reverse transcription using 1 µg of total RNA (Promega Reverse Transcription System, Promega, USA). Quantitative-RT-PCR was performed using SYBR Select Master Mix (Applied Biosystems, Life Technologies, USA) and operated by Applied Biosystems 7500 Real Time PCR System (Applied Biosystems, Life Technologies, USA). The data were normalized by *β-actin*. Primers used are listed in Table S2.

### Gene knockdown and overexpression

The vectors of either for knockdown or overexpression of *Hdac6* was respectively mixed with trypan blue solution (vector/trypan blue solution: 10/1) and then injected into isolated 1 dpp mouse ovaries using glass pipettes under a stereomicroscope. After the ovaries were injected with the blue liquid, electrotransfection was performed by the application of three 5-ms-long quasi-square pulses at a pulse-field strength of up to 30 V/cm. The *Hdac6* knockdown vector was constructed by cloning *Hdac6*-shRNA with sequence: GGTACTTCCCATCGCCTATGA. The *Hdac6* overexpression vector was constructed by cloning the open reading frame of *Hdac6* into the pCMV-C-His (D2650, Beyotime Biotechnology, China). Golden star t6 super PCR mix were purchased from Beijing Tsingke Co., Ltd. All of the constructs were verified by sequencing. Primers are listed in Table S3.

### Statistical analysis

All experiments were repeated at least three times. The values are presented as the means ± SEM. The data were analyzed by either *t* test or ANOVA, and considered statistically significant at *P* < 0.05.

## Results

### HDAC6 is indispensable for preventing primordial follicle activation in vitro

**Fig. 1:**
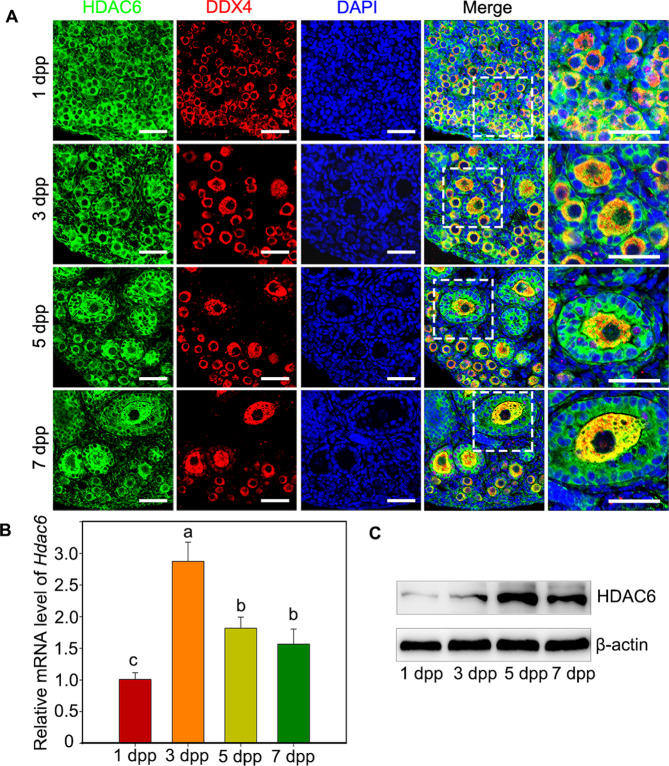
The expression pattern of HDAC6 in the neonatal mouse ovaries. **A** Cellular localization of HDAC6 in newborn ovaries. Newborn mice ovaries were stained for HDAC6 (green) and the oocyte specific marker DDX4 (red) at the indicated time points. The nuclei were counter-stained by DAPI (blue). HDAC6 was mainly localized to the cytoplasm of both oocytes and granulosa cells in either primordial follicles or growing follicles. **B** qRT-PCR assay showed that the expression level of *Hdac6* mRNA was increased from 1 dpp to 5 dpp and decreased on 7 dpp. **C** Western blotting assay showed that the level of HDAC6 protein was increased from 1 dpp to 5 dpp and decreased on 7 dpp as well. The experiments were repeated at least three times. And representative images are shown. The data are presented as the means ± SEM. Scale bars: 50 μm.

To explore the potential role of HDAC6 in either sustaining the dormancy or activation of primordial follicle, immunofluorescence staining was firstly performed to detect the cellular localization and expression dynamics of HDAC6 in newborn and adult mouse ovaries. It showed that HDAC6 was predominantly localized to the cytoplasm of oocytes and granulosa cells in newborn and adult mice ovaries (Figs. 1A and S1).

We found that the signal of HDAC6 was homogenous stronger in the majority of primordial follicles, but was relatively weaker in some primordial follicles, which may be on the way to be activated. This phenomenon is observed in both ovaries of adult and newborn mice unanimously (Figs. S1 and S2). The statistics results show that 3.5 ± 0.2% of primordial follicles with weaker HDAC6 expression are presented in adult ovaries, while 4 ± 0.4% of primordial follicles with weaker HDAC6 expression are presented in 5 dpp ovaries (Fig. S3).

In order to clarify whether the primordial follicles with weaker HDAC6 signal are being activated, the 5 dpp ovaries were stained for HDAC6 and Foxo3a in adjacent serial sections. The results showed that over 60% of primordial follicles with weaker expression of HDAC6 were simultaneously the ones whose FOXO3a was located in the cytoplasm (Fig. R6). The results suggest that 65.1 ± 5.3% of primordial follicles with weaker HDAC6 signal are being activated.

Then the qRT-PCR and western blotting analyses of whole ovary samples revealed that the expressions of both the mRNA and protein of HDAC6 increase significantly along with the establishment of the primordial follicle pool at 3 dpp, and retain at a relative higher level from 5 dpp to 7 dpp, during which time primordial follicle activation is generally initiated (Fig. 1B, C).

To further determine the expression pattern of HDAC6 in primordial follicles from 1 dpp to 7 dpp, a model of ovarian cortex fragments isolated from the intact ovary was established. We noticed that only primordial follicles were found in the isolated cortical fragments in 1 dpp–7 dpp ovaries (Figs. S5–S9). The western blotting results showed that the HDAC6 expression decreased from 1 dpp and then maintained at a relatively weaker level from 3 dpp to 7 dpp in ovarian cortex fragments. Interestingly, the general protein level of HDAC6 in 5 dpp whole ovary was higher than in 3 dpp–7 dpp ovarian cortex fragments (Fig. S10). Collectively, these results suggest that the expression of HDAC6 in primordial follicles is homogenous, whereas the expression of HDAC6 becomes weaker in some primordial follicles that were to be activated. These results indicated that HDAC6 plays a potential role in primordial follicle activation versus quiescent.

**Fig. 2:**
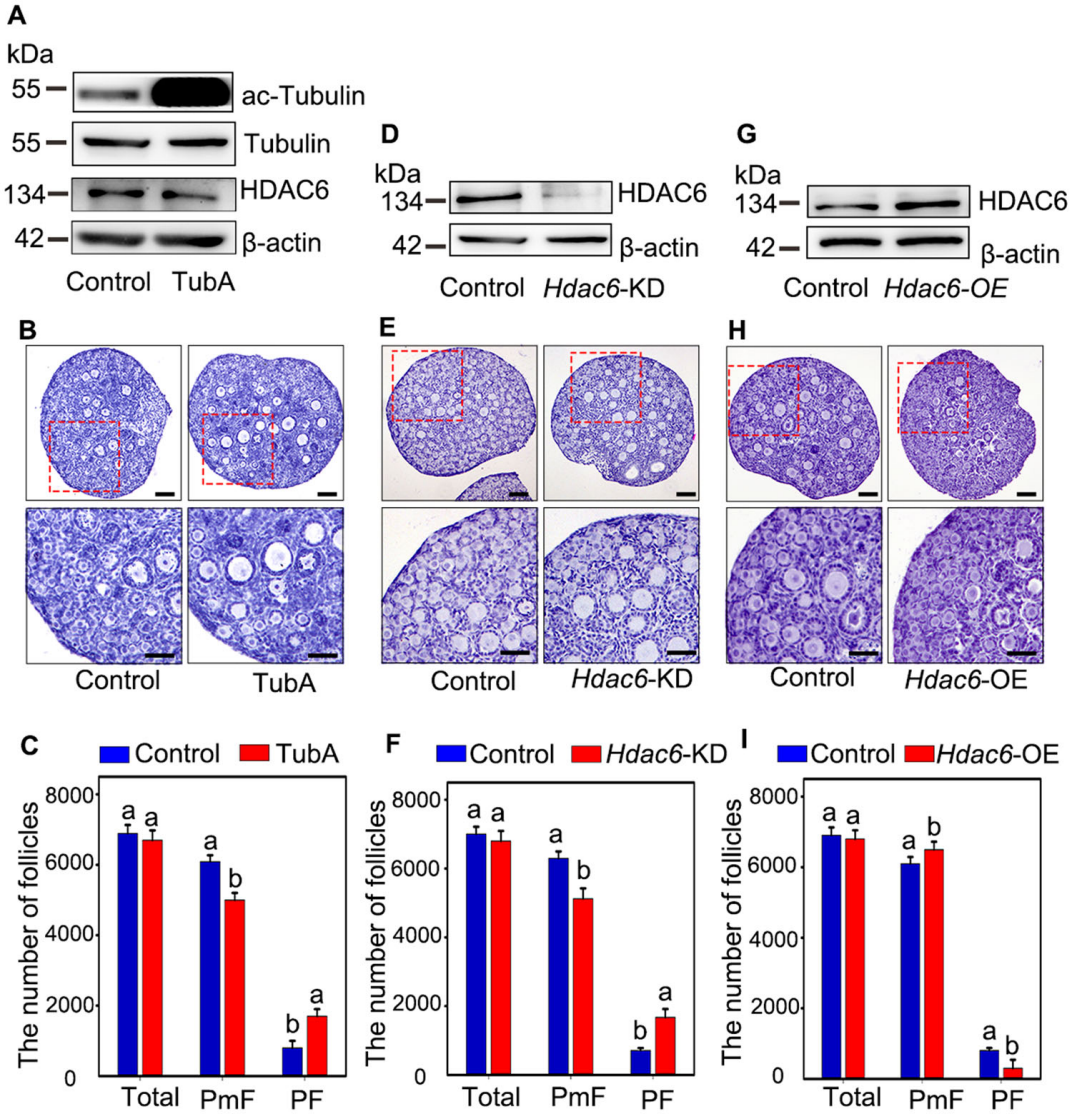
HDAC6 is involved in the primordial follicle activation. **A** Inhibition efficiency analysis of TubA. The 2 dpp ovaries were cultured with or without TubA for 2 days. The ac-Tubulin was increased while HDAC6 was decreased in TubA treatment. ac-Tubulin: acetylated alpha-tubulin. **B**, **C** The histological analysis and follicle counting results showed that inhibition of HDAC6 expression by TubA accelerated primordial follicles activation without affecting the number of total follicles. The 2 dpp ovaries were cultured with or without TubA for 3 days. **D** Knockdown efficiency analysis of *Hdac6*-KD. The 1 dpp ovaries were transfected with *Hdac6*-KD vector and cultured for additional 3 days. The level of HDAC6 was significantly decreased in *Hdac6*-KD treatment. **E**, **F** The histological analysis and follicle counting results showed that knock down of *Hdac6* expression accelerated primordial follicles activation without affecting the number of total follicles. The 1 dpp ovaries were transfected with *Hdac6*-KD vector and cultured for 4 days to assess the influence of RNAi on follicle development. **G** Overexpression efficiency analysis of *Hdac6*-OE. The 1 dpp ovaries were transfected with *Hdac6*-OE vector and cultured for 3 days. The level of HDAC6 was significantly increased in *Hdac6*-OE treatment. **H**, **I** The histological analysis and follicle counting results showed that overexpression of *Hdac6* retarded the activation of primordial follicles without affecting the number of total follicles. The 1 dpp ovaries were transfected with *Hdac6*-OE vector and cultured for 4 days to assess the influence of overexpression of *Hdac6* on follicle development. The experiments were repeated at least three times. And representative images are shown. The letters indicate there is a significant difference between the control and the specific treatment group. The data are presented as the means ± SEM. Scale bars: 50 μm.

To study the function of HDAC6 in primordial follicle in newborn mouse ovaries, an in vitro culture model was established. The histological and statistical analysis results showed that the ovaries cultured in vitro develop normally as the morphology as well as the numbers of different stage of follicles and granulosa cells were consistent with the simultaneously developed ovaries in vivo (Fig. S11). Based on this model, 2 dpp ovaries were cultured with TubA, one of the inhibitors of HDAC6, for 2 days. The results showed that acetylated alpha-tubulin was increased and the level of HDAC6 was decreased compared to the control (Fig. 2A). Based on these findings, the treated ovaries were furtherly cultured for 3 days before histological analysis. The results showed that more growing follicles were observed in TubA treatment compared to the control, while the number of total follicles within each ovary was comparable to the control (Fig. 2B, C). These results implied that HDAC6 participates in preventing primordial follicles from premature activation in vivo.

To further confirm the function of HDAC6 in regulating primordial follicle development, both *Hdac6*-shRNA (*Hdac6*-KD) vector and *Hdac6*-overexpression (*Hdac6*-OE) vector were respectively constructed, and injected into 1 dpp ovaries respectively. The injected ovaries were cultured for 3 days, the results showed that the endogenous HDAC6 protein levels were either efficiently down-regulated by *Hdac6*-KD, or up-regulated in *Hdac6*-OE group, respectively (Fig. 2D, G). Then, the injected ovaries were cultured for 4 days, the histological analysis showed that more growing follicles were observed in *Hdac6*-KD group and fewer growing follicles were observed in *Hdac6*-OE group, respectively (Fig. 2E, H). The quantitative results further confirmed the phenotype observed in Figs. 2F, I. These results indicated that downregulating the level of HDAC6 was necessary for mice primordial follicles activation in vitro.

### HDAC6 prevents primordial follicle activation through inhibiting mTOR signaling pathway

**Fig. 3:**
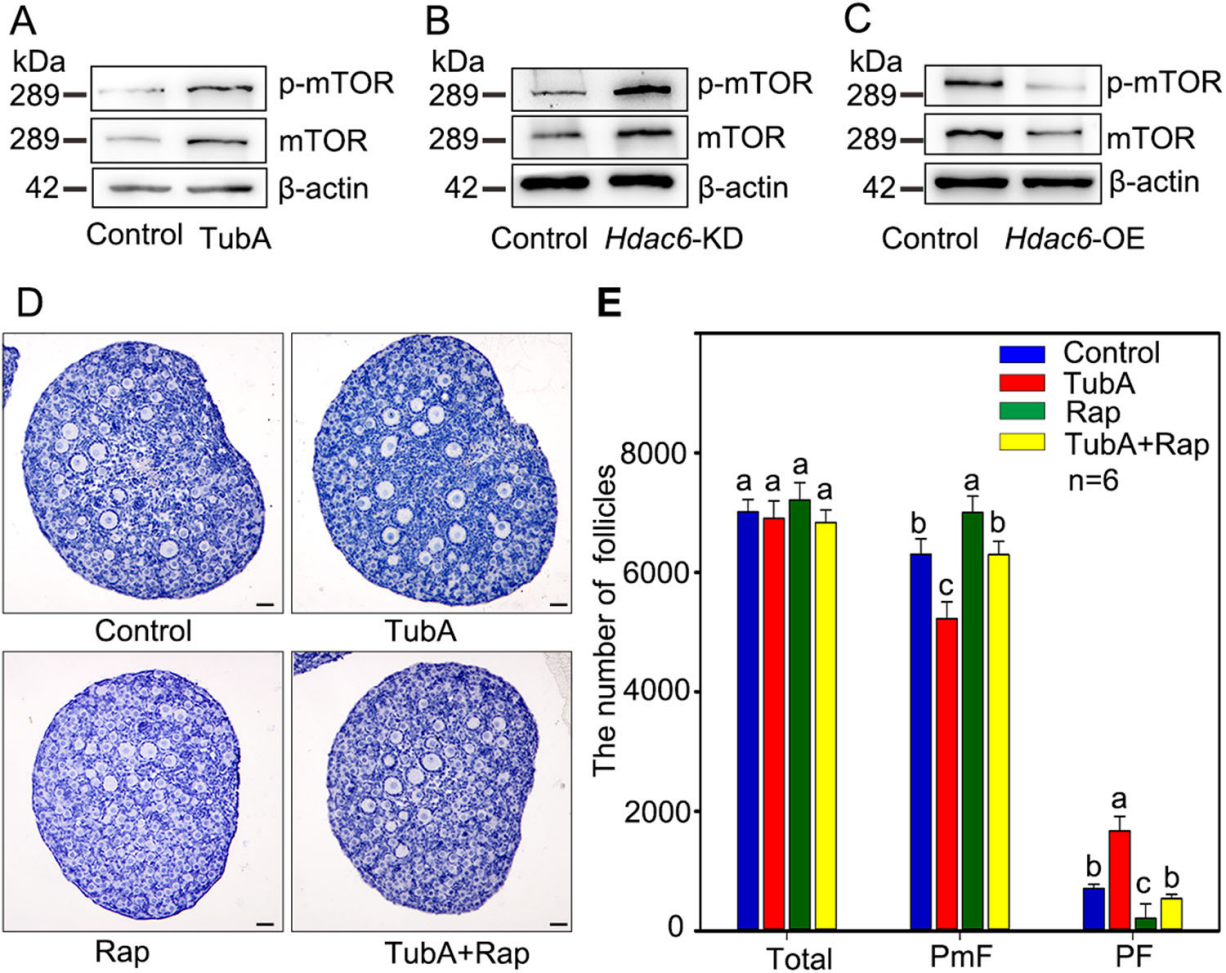
HDAC6 is responsible for primordial follicle activation by regulating mTOR signaling pathway. **A**–**C** The mTOR signaling pathway in the ovaries was affected by either inhibiting or elevating the level of HDAC6. Western blotting results showed that both total-mTOR and p-mTOR were increased in the TubA group or *Hdac6*-KD group compared with those in the respective controls. On contrary, *Hdac6*-OE treatment decreased the levels of both total-mTOR and p-mTOR compared with the control. **D**, **E** The histological analysis and counting results showed that the primordial follicle activation was reversed in TubA plus Rap treatment compared with TubA treatment alone. The experiments were repeated at least three times. And representative images are shown. The data are presented as the means ± SEM. Scale bars: 50 μm.

To investigate how decreased HDAC6 participates in primordial follicle activation, the changes of the mTOR signaling pathway were detected. Before examination, 2 dpp ovaries were cultured with or without TubA for 2 days. The western blotting results showed that both TSC1 and TSC2 were unchanged (Fig. S12) whereas the levels of both total and phosphorylated mTOR which are the downstream molecules of TSCs, were up-regulated in TubA treatment (Fig. 3A). To further confirm the regulation effect of decreased HDAC6 on mTOR, the 1 dpp ovaries were either injected with *Hdac6*-KD or *Hdac6*-OE vector respectively and cultured for additional 3 days. Western blotting results showed that the levels of both total and phosphorylated mTOR were either up-regulated in *Hdac6*-KD treatment or down-regulated in *Hdac6*-OE treatment, respectively (Fig. 3B, C). These results suggested that HDAC6 may be pivotal for balancing primordial follicle between dormancy and activation through influencing the mTOR signaling pathway.

To further confirm the above conclusion, Rapamycin (Rap), the specific inhibitor of mTOR, was used to verify if it could attenuate the expression of mTOR induced by TubA treatment. For this purpose, 2 dpp ovaries were cultured with either TubA or Rap for 3 days before examination. The histological analysis and whole ovary counting results showed that fewer growing follicles were observed in TubA plus Rap treatment than those in the TubA treatment (Fig. 3D–F). Inhibition of mTOR by Rap reversed the follicular development caused by TubA. These results suggested that HDAC6 in the primordial follicles of new born ovaries prevent follicles from premature activation.

### Downregulation of HDAC6 is necessary for activating PI3K-AKT pathway in oocytes in vitro

**Fig. 4:**
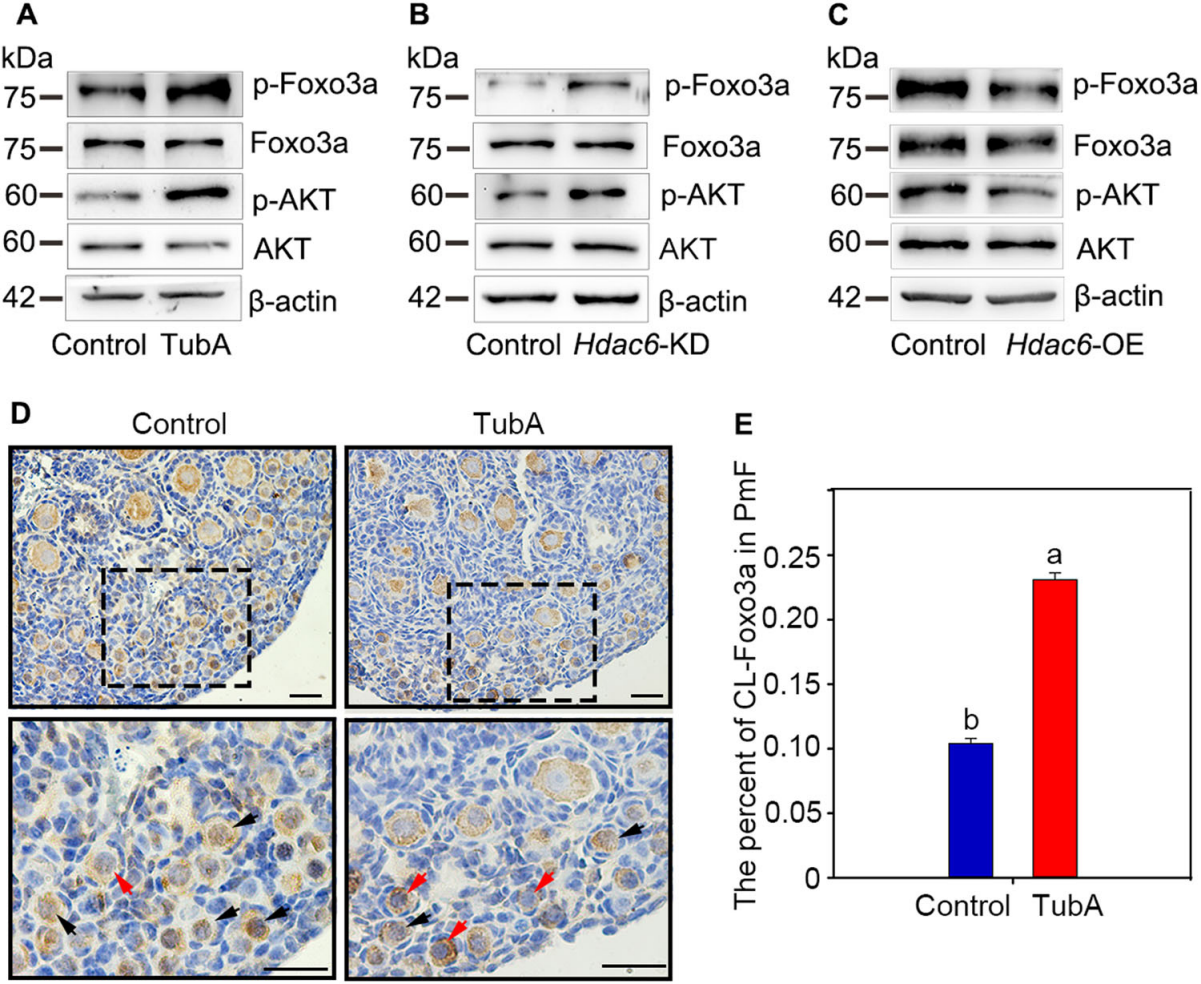
HDAC6 participates in the maintenance and activation of PI3K signaling. **A**–**C** Western blotting results showed that both p-Foxo3a and p-AKT were increased in either TubA or *Hdac6*-KD group compared with those in the controls, respectively. On contrary, the levels of both p-Foxo3a and p-AKT were down-regulated by *Hdac6*-OE treatment compared with the control. **D** The immunostaining stain showed that the cytoplasmic translocation of Foxo3 was increased in primordial follicles after treated with Tub A. Red arrow indicates cytoplasm Foxo3a. Black arrow indicates nucleus Foxo3a. **E** The statistical results showed that the cytoplasmic translocation of Foxo3 was increased in primordial follicles after treated with TubA. At least 500 primordial follicles were counted in each group. Scale bars: 50 μm.

To study the potential relationship between down-regulated HDAC6 and the PI3K pathway within oocytes of primordial follicles, 2 dpp ovaries were cultured with or without TubA for 2 days. Western blotting results showed that the total levels of either AKT or Foxo3a were unchanged, while the levels of both phosphorylated AKT and Foxo3a were up-regulated in TubA treatment (Fig. 3A). At the same time, the conclusion was further confirmed by results applying *Hdac6-*KD and *Hdac6-*OE, respectively (Fig. 4B, C). In addition, immunofluorescence staining and quantitative results showed that Foxo3a, which shuttled from the nucleus to the cytoplasm, was increased in TubA treated ovaries (Fig. 4D). These results indicated that inhibition of HDAC6 expression in mice ovaries was indispensable for the activation of PI3K pathway.

### Down-regulated HDAC6 in new born ovaries governed primordial follicle activation through KITL-KIT in vitro

According to the existed hypothesis, it is somatic cell that initiates primordial follicle activation. That is, the up-regulated mTORC1 signaling in pre-granulosa cells triggers the awakening of dormant oocytes through secreting KIT ligand (KITL). The coupling between mTORC1-KITL in pfGCs and KIT-PI3K signaling in oocytes awakens primordial follicle finally^14^. In line with this, we therefore further examined the signal cascade within the oocytes in our TubA supplementing assays. The results showed that in addition to the activation of mTOR, the PI3K-AKT pathway activity was increased in TubA treatment accordingly.

Therefore, we hypothesized that down-regulated HDAC6 may be involved in the activation of primordial follicles by activating mTOR and improving pfGCs differentiation into granulosa cells followed by activating oocytes. To prove this hypothesis, the following assays were performed.

**Fig. 5:**
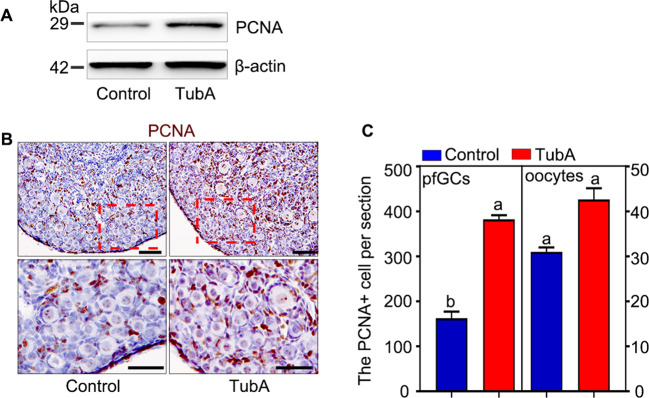
HDAC6 regulates the proliferation of primordial follicle granulosa cell. **A** The western blotting results showed PCNA was increased after 2 dpp ovaries was cultured with TubA for 1 day. **B** Immunostaining stain of PCNA results showed that the growth of pfGCs in primordial follicles was increased compared with the control after 2 dpp ovaries were cultured with or without TubA for 1 day. **C** PCNA counting results showed that PCNA positive signaling was increased in pfGCs after with TubA compared with the control. The experiments were repeated at least three times. And representative images are shown. Scale bars: 50 μm.

On the one hand, 2 dpp ovaries were cultured for 1 day with TubA and collected to examine the growth of ovaries. The western blotting results showed that the expression of PCNA protein within ovaries was up-regulated in TubA treatment (Fig. 5A), which implied that the growth of the whole ovaries was accelerated by TubA. Immunofluorescence staining and counting of PCNA positive cell results also showed that the PCNA positive signal was significantly increased in pfGCs (Fig. 5B, C). These results suggested that the growth of ovaries, especially the growth of pfGCs, was accelerated by TubA.

**Fig. 6:**
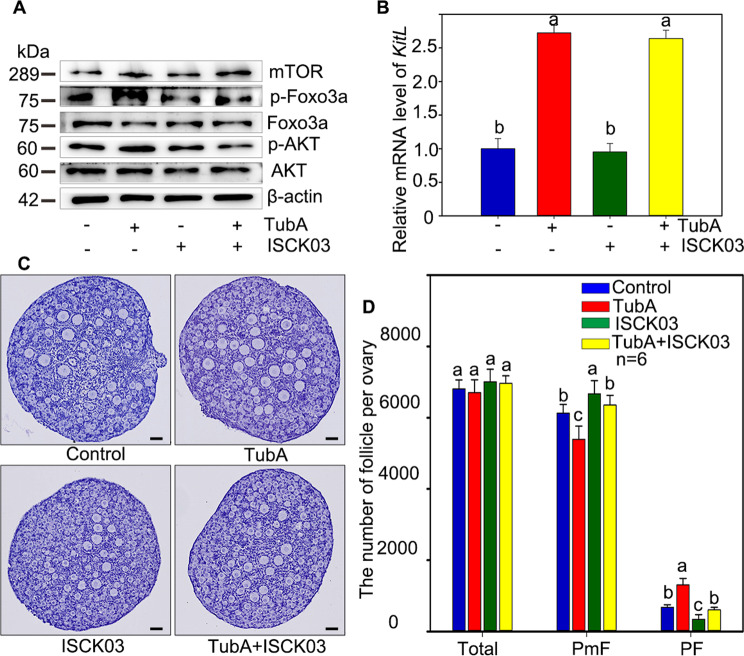
HDAC6 regulated primordial follicle activation through mTOR-KITL signaling. **A** The western blotting results showed the protein expression of mTOR, p-Foxo3a, Foxo3a, p-AKT, and AKT in TubA group, ISCK03 group and TubA plus ISCK03 group, respectively. **B** The *Kitl* mRNA relative level in TubA group, ISCK03 group and TubA plus ISCK03 group, respectively. **C**, **D** The histological analysis and counting results showed that the primordial follicle activation was reversed in TubA plus ISCK03 treatment compared with TubA treatment. The experiments were repeated at least three times. And representative images are shown. The data are presented as the means ± SEM. Scale bars: 50 μm.

On the other hand, we blocked the communication network between pfGCs and oocyte by ISCK03, one of the KIT inhibitors, in TubA assay. The results showed that both p-AKT and p-Foxo3a were decreased (Fig. 6A, B) despite that mTOR and KITL were increased in TubA plus ISCK03. In which case, the primordial follicle activation was retarded according to the histomorphology and statistical results (Fig. 6C, D). The results suggested that HDAC6 controls primordial follicle activation through KITL-KIT signaling.

## Discussion

Primordial follicle pool established perinatally determines the length of the female reproductive lifespan. In this study, we found that HDAC6, one of the epigenetic modification proteins, is pivotal for preserving fertility of mice through inhibiting mTOR signaling in pfGCs of mice primordial follicles. Therefore, the detailed findings from this study supplied additional proofs to deeper understand the underlined mechanisms of primordial follicle quiescent.

Our study approved that the expression level of HDAC6 in primordial follicles is heterogeneous within the ovary. Because those primordial follicles with weaker HDAC6 expression are being activated ones while those with stronger HDAC6 are quiescent ones, it is possible that the decreased level of HDAC6 in primordial follicles indicates the fate of the follicles is activation. During the process, the decline of HDAC6 may be transient because the levels of HDAC6 elevates in primary follicles soon. According to previous studies, short-term activation treatment with one of the activators of either AKT, CDC42, or SIRT1 promotes the activation of mouse primordial follicles in vitro^9,11,12,17,31,32^. Therefore, the transition of primordial follicles from the dormant state to the activated state may be a very short process.

The activation of primordial follicles is a relatively balancing process. Properly controlling the speed of primordial follicle activation prolongs the reproductive life of the ovaries^33–35^. According to previous report, the ovarian reproductive lifespan was extended in *Hdac6* overexpression mice ovary^18^. It is assumed that overexpression of *Hdac6* may retard the activation of primordial follicles and slow down the rate of follicle consumption, which prolongs the lifespan of mice ovaries. Although our study has uncovered the independent role of HDAC6 in primordial follicle activation process in mice ovaries in vitro, whether HDAC6 regulates the activation speed of primordial follicles to extend reproductive life requires further study.

Interestingly, according to this study, the action of HDAC6 on sustaining primordial follicle dormancy in mice seems to overlap the findings of TGF-β according to our previous study^13^. We noticed that not only the expression pattern of TGF-β and HDAC6 in new born mice ovaries are similar to each other, but the level of TGF-β, as well as its downstream key protein, namely the activated SMAD3, decreased in response to inhibitor of HDAC6. According to Wang et al.^13^, down-regulated TGF-β results in primordial follicle activation. However, whether HDAC6 is the upstream or downstream molecular of TGF-β within cells is controversial based on specific cell types been researched^36,37^. Besides, although we have proved that high level of E-cadherin in oocytes, and lower level of Sirt1in both oocytes and pfGCs, are also positive molecules to inactivate primordial follicles, we did not find any relationship between HDAC6, SIRT1, and E-cadherin so far (data not shown), implying that there may have multiple signal pathways existing in primordial follicle. Additional studies are needed to clarify the complex relationship between these molecules.

This study re-emphasizes the importance of epigenetic modification in sustaining the primordial follicle pool in vivo. Growing evidences from ours and others have proved that the development of either germ cells or early embryos are under controlled by epigenetic modification proteins, like histone deacetylases including HDAC1, HDAC2, HDAC3, SIRT1, HDAC6, as well as histone demethylase like LSD1^38–45^. We have proved that the common target protein of both SIRT1 and HDAC6 in pfGCs is unexceptionally mTOR^12^. On the other hand, since the present hypothesis referring to primordial follicle activation underlines the importance of the communication between the somatic cells and oocytes within follicle^14^, our study has supplied additional proofs to support the theory soundly. Our study also indicates that there are multiple signal pathways governing the process of activating primordial follicles, which worth further study based on more complicated in vitro and in vivo models.

The levels of activated mTOR as well as PI3K proteins determine the fate of primordial follicles, as has been proved by this study and others. First, upregulation of mTOR in either pfGCs or oocyte accelerates primordial follicle activation^46,47^. Conditional knockout of mTOR in oocytes of primordial follicle causes defective follicular development leading to progressive degeneration of oocytes^48^, whereas conditional knockout of *Tsc1*, the negatively regulator of mTOR, in either oocytes or pfGCs of primordial follicles resulted in systematic premature activation of primordial follicle pool^14,31^. Second, increasing evidences from others and ours have indicated the importance of the activity of PI3K signaling pathway, in determining if oocytes within primordial follicles could be activated, no matter it is activated by signals from oocyte itself or from granulosa cells of the primordial follicle^8,9,11,12,15^. The activity of AKT in response to inhibition of HDAC6 seems to be cell type dependent^36^. In this study, HDAC6 inhibition results in increased AKT kinase activity indirectly in newborn mice ovary through activated mTOR-KITL pathway in pfGCs. Further studies are needed to clarify the exact factors within the niche of primordial follicles which are the key signals for sustaining the quiescent of primordial follicles before a clearer model of primordial follicle sustaining be figured out.

In vitro activation (IVA) of primordial follicle technique based on above mentioned theory has been established to help solve the problems of POF patients recently^31^. The main target for the tech is to inhibit the activity of PTEN or activate PI3K in oocytes^31^. From this study, we have provided an alternative target, the HDAC protein in pfGCs, for activating mTOR-KIT-PI3K signaling cascades between somatic cells and oocytes within individual primordial follicle. However, although we have proved that activating SIRT1 contributes to higher IVA efficacy in mice, the reported efficiency of IVA in human being is quite low so far^16^, implying that there might have other unknown signal transduction pathways responsible for dynamic regulation of primordial follicles fate.

In summary, HDAC6 in pfGCs of primordial follicle may play a key role in sustaining primordial follicle at dormant state by modulating mTOR-KITL signal. These findings provided further proofs demonstrating that epigenetic modification participates in homeostatic of primordial follicle reserve.

## Supplementary information

Supplemental Figure Legend

Supplemental Tables

Figure S1

Figure S2

Figure S3

Figure S4

Figure S5

Figure S6

Figure S7

Figure S8

Figure S9

Figure S10

Figure S11

Figure S12
