# Supplementary material for: HDAC6 regulates primordial follicle activation through mTOR signaling pathway
Source: Cell Death Dis. 2021 May 29;12(6):559. doi: 10.1038/s41419-021-03842-1 (PMC8164630; doi:10.1038/s41419-021-03842-1)
Supplement: Supplementary file 1 — Supplemental Figure Legend [file 41419_2021_3842_MOESM1_ESM.doc]

**Fig. S1 The expression of HDAC6 in primordial follicles from adult ovaries is heterogeneous**

Adult mice ovaries were stained for HDAC6 (green). The nuclei were stained by DAPI (blue). **A** (*Upper*) Primordial follicles showed high HDAC6 expression in adult ovaries. The red arrow indicates the primordial follicles (red 1^#^, 2^#^ and 3^#^). (*Lower*) Magnified view of the area indicated by the red arrow in the *Upper* image. **B** (*Upper*) Primordial follicles showed low HDAC6 expression in adult ovaries. The white arrow indicates the primordial follicle (white 1^#^, 2^#^ and 3^#^). (*Lower*) Magnified view of the area indicated by the white arrow in the *Upper* image. PmF: primordial follicle. Scale bars: 10 μm.

**Fig. S2 The expression of HDAC6 in primordial follicles from newborn ovaries is heterogeneous**

The 5 dpp mice ovaries were stained for HDAC6 (green) and the oocyte marker DDX4 (red). The nuclei were stained by DAPI (blue). (*Upper*) Primordial follicles showed heterogeneous HDAC6 expression in 5 dpp ovaries. (*Lower*) Magnified view of the white circle area in the *Upper* image. Scale bars: 10 μm.

**Fig. S3 The percent of high and low HDAC6 expression primordial follicle in adult ovaries and newborn ovaries**

The whole ovary counting analyses the primordial follicles with high HDAC6 expression and high HDAC6 expression. At least 6 ovaries were counted in every group. The results showed that 3.5 ± 0.2% of primordial follicles with low HDAC6 expression in adult ovaries, 4.1 ± 0.4% of primordial follicles with low HDAC6 expression in 5 dpp ovaries.

**Fig. S4 About 65% of primordial follicles with weaker HDAC6 signal are being activated**

**A** The 5 dpp ovaries were sectioned and stained for HDAC6 (green) or Foxo3a (red) in adjacent sections. Nuclei were dyed with a DAPI (blue). The results showed that Foxo3a began to be transported out of the nuclear in some primordial follicles with weaker HDAC6 expression. Foxo3a was transported out of the nuclear in primary follicles. Scale bar: 10 μm. **B** The whole ovary counting analyses the cellular location of Foxo3a in primordial follicles with weaker HDAC6 expression. At least 6 ovaries were counted in every group. The results show that in 65.1 ± 5.3% of primordial follicles with weaker HDAC6 expression, Foxo3a was expressed at the cytoplasm in these primordial follicles. In the other primordial follicles with weaker HDAC6 expression, Foxo3a was expressed in the nucleus. CL-Foxo3a: cytoplasm Foxo3a; NL-Foxo3a: nucleus Foxo3a.

**Fig. S5 The histological analysis showed that 1 dpp whole ovaries as well as the cortex fragments of these ovaries contain only primordial follicles**

**A** Hematoxylin staining of 1 dpp whole ovary. Scale bar: 100 μm. **B** Magnified view of 1 dpp whole ovary. Scale bar: 100 μm. **C** Magnified view of the red box enclosed area in the B image. Of which, all follicles within the ovaries are primordial follicles. Scale bar: 25 μm. **D** Magnified view of the green box enclosed area in the B image. Of which, all follicles within the ovaries are primordial follicles. Scale bar: 25 μm. **E** Hematoxylin staining of 1 dpp ovarian cortex fragment. Scale bar: 100 μm. **F** Magnified view of the red box enclosed area in the E image. Of which, all follicles within the ovaries are primordial follicles. Scale bar: 25 μm. **G** Magnified view of the green box enclosed area in the E image. The all follicles are primordial follicles. Scale bar: 25 μm.

**Fig. S6 The histological analysis showed that 3 dpp whole ovaries contain both primordial follicles and primary follicles, whereas 3 dpp ovarian cortex fragments contain only primordial follicles.**

**A** Hematoxylin staining of 3 dpp whole ovary. Scale bar: 100 μm. **B** Magnified view of ovary 3 dpp whole ovary. Scale bar: 100 μm. **C** Magnified view of the red box enclosed area in the B image. The presented follicle is primary follicle. Scale bar: 25 μm. **D** Magnified view of the green box enclosed area in the B image. These follicles are primordial follicles. Scale bar: 25 μm. **E** Hematoxylin staining of 3 dpp ovarian cortex fragment. Scale bar: 100 μm. **F** Magnified view of the red box enclosed area in the E image. These follicles are primordial follicles. Scale bar: 25 μm. **G** Magnified view of the green box enclosed area in the E image. These follicles are primordial follicles. Scale bar: 25 μm

**Fig. S7 The histological analysis showed that 5 dpp whole ovaries contain both primordial follicles and primary follicles, whereas 5 dpp ovarian cortex fragments contain only primordial follicles.**

**A** Hematoxylin staining of 5 dpp whole ovary. Scale bar: 100 μm. **B** Magnified view of ovary 5 dpp whole ovary. Scale bar: 100 μm. **C** Magnified view of the red box enclosed area in the B image. The presented follicle is primary follicle. Scale bar: 25 μm. **D** Magnified view of the green box enclosed area in the B image. These follicles are primordial follicles. Scale bar: 25 μm. **E** Hematoxylin staining of 5 dpp ovarian cortex fragment. Scale bar: 100 μm. **F** Magnified view of the red box enclosed area in the E image. These follicles are primordial follicles. Scale bar: 25 μm. **G** Magnified view of the green box enclosed area in the E image. These follicles are primordial follicles. Scale bar: 25 μm.

**Fig. S8 The histological analysis showed that 7 dpp whole ovaries contain primordial follicles and primary follicles, whereas 7 dpp ovarian cortex fragments contain only primordial follicles.**

**A** Hematoxylin staining of 7 dpp whole ovary. Scale bar: 100 μm. **B** Magnified view of ovary 7 dpp whole ovary. Scale bar: 100 μm. **C** Magnified view of the red box enclosed area in the B image. The presented follicle is primary follicle. Scale bar: 25 μm. **D** Magnified view of the green box enclosed area in the B image. These follicles are primordial follicles. **E** Hematoxylin staining of 7 dpp ovarian cortex fragment. Scale bar: 100 μm. **F** Magnified view of the red box enclosed area in the E image. These follicles are primordial follicles. Scale bar: 25 μm. **G** Magnified view of the green box enclosed area in the E image. These follicles are primordial follicles. Scale bar: 25 μm.

**Fig. S9 The percentages of different stage follicles in whole ovaries and ovarian cortex fragments from 1 dpp to 7 dpp**

The follicle counting analyses in whole ovary and ovarian cortex fragment from 1 dpp to 7dpp. At least 500 follicles were counted in every group. **A** All follicles are primordial follicles in 1 dpp whole ovary and 1 dpp ovarian cortex fragment. **B** The 3 dpp whole ovary contains 98.0 ± 10.2% primordial follicles plus 2.0 ± 0.2% primary follicles. All follicles are primordial follicles in 3 dpp ovarian cortex fragment. **C** The 5 dpp whole ovary contains 95.8 ± 11.4% primordial follicles plus 4.2 ± 0.5% primary follicles. The 5 dpp ovarian cortex fragment contains 99.4 ± 10.2% primordial follicles plus 0.6 ± 0.03% primary follicles. **D** The 7 dpp whole ovary contains 93.7 ± 10.2% primordial follicles plus 6.3 ± 0.7% primary follicles. The 7 dpp ovarian cortex fragment contains 99.8 ± 10.2% primordial follicles plus 0.2 ± 0.01% primary follicles.

**Fig. S10 The expression pattern of HDAC6 in the neonatal mouse ovarian cortex fragments**

The ovarian cortex fragments from 1 dpp to 7dpp were isolated. The 5 dpp whole ovaries are treated as positive control. Western blotting results showed that the expression of HDAC6 decreased from 1 dpp and then maintained at a relatively low level from 3 dpp to 7 dpp in ovarian cortex fragments. The expression level of HDAC6 in whole ovaries at 5 dpp was significantly higher than that in 3 dpp, 5 dpp and 7 dpp ovarian cortex fragments.

**Fig. S11 Model of newborn mice ovaries cultured *in vitro* was established**

**A** Immunofluorescence staining and histological analyses of ovaries at 5 dpp *in vivo* and 2 dpp followed by 3 days of *in vitro* culture (equal to 5 dpp *in* *vivo*). Oocytes were stained with DDX4 (green), pfGCs and GCs were stained with FOXL2 (red). The nuclei were stained by DAPI (blue). **B** The follicle counting results of total follicles, primordial follicles and primary follicles. **C** The somatic cell counting results of pfGCs and GCs. Total: total follicle; PmF: primordial follicle; PF: primary follicle; pfGCs: primordial follicles granulosa cells; GC: granulosa cells.

**Fig. S12 HDAC6 did not affect TSC1/2 in cultured newborn mice**

Western blot results showed that TSC1 and TSC2 was unchanged in TubA group compared with the control.
