# Supplementary material for: HDAC6 regulates primordial follicle activation through mTOR signaling pathway
Source: Cell Death Dis. 2021 May 29;12(6):559. doi: 10.1038/s41419-021-03842-1 (PMC8164630; doi:10.1038/s41419-021-03842-1)
Supplement: Supplementary file 2 — Supplemental Tables [file 41419_2021_3842_MOESM2_ESM.doc]

**Supplemental Tables**

**Table S1 Antibodies**

| **Antibodies** | **Vendors; Cat. No.** | **Source** | **Dilution/Applications** |
| --- | --- | --- | --- |
| HDAC6 | Beyotime Biotechnology; AH395 | Rabbit | 1:1000 (WB); 1:200 (IF) |
| AKT | Cell Signaling Technology; 4685 | Rabbit | 1:1000 (WB) |
| p-AKT | Beyotime Biotechnology; AA331 | Rabbit | 1:1000 (WB) |
| mTOR | Cell Signaling Technology; 2983 | Rabbit | 1:500 (WB) |
| p-mTOR | Cell Signaling Technology; 5536 | Rabbit | 1:500 (WB) |
| DDX4 | Abcam; ab27591 | Mouse | 1:200 (IF) |
| FOXL2 | Nouvs; NB100-1277 | Goat | 1:500 (IF) |
| Foxo3a | Abcam; ab12162 | Rabbit | 1:1000 (WB); 1:200 (IF) |
| p-Foxo3a | Santa Cruz; Sc-12357 | Rabbit | 1:500 (WB) |
| PCNA | Abcam; ab18197 | Mouse | 1:1000 (WB); 1:200 (IHC) |
| β-actin | Cwbiotech; CW0096M | Mouse | 1:5000 (WB) |
| TSC1 | Cell Signaling Technology; 6935 | Rabbit | 1:1000 (WB) |
| TSC2 | Cell Signaling Technology; 4308 | Rabbit | 1:1000 (WB) |
| Alexa Fluor® 488 | Thermo Scientific; A-11034 | Donkey | 1:200 (IF) |
| Alexa Fluor® 555 | Thermo Scientific; A-21127 | Donkey | 1:200 (IF) |
| DAPI | Merck; D9542 |  | 1:500 |

## Table S2 Primers real-time PCR

| **Genes** | **Primers** |
| --- | --- |
| *β-actin-F* | GTGACGTTGACATCCGTAAAGA |
| *β-actin-R* | GCCGGACTCATCGTACTCC |
| *Hdac6-F* | TCCACCGGCCAAGATTCTTC |
| *Hdac6-R* | CAGCACACTTCTTTCCACCAC |

## Table S3 Primers for *Hdac6* CDS

| **Gene** | **Primers** |
| --- | --- |
| *Hdac6-F-* Xho I | CCGCTCGAGATGACCTCCACCGGCCAAGATT |
| *Hdac6-R-*Not I | ATTTGCGGCCGCTTAGTGTGAGTGGGGCAT |
