# Supplementary figures and images for: HDAC6 regulates primordial follicle activation through mTOR signaling pathway
Source: Cell Death Dis. 2021 May 29;12(6):559. doi: 10.1038/s41419-021-03842-1 (PMC8164630; doi:10.1038/s41419-021-03842-1)

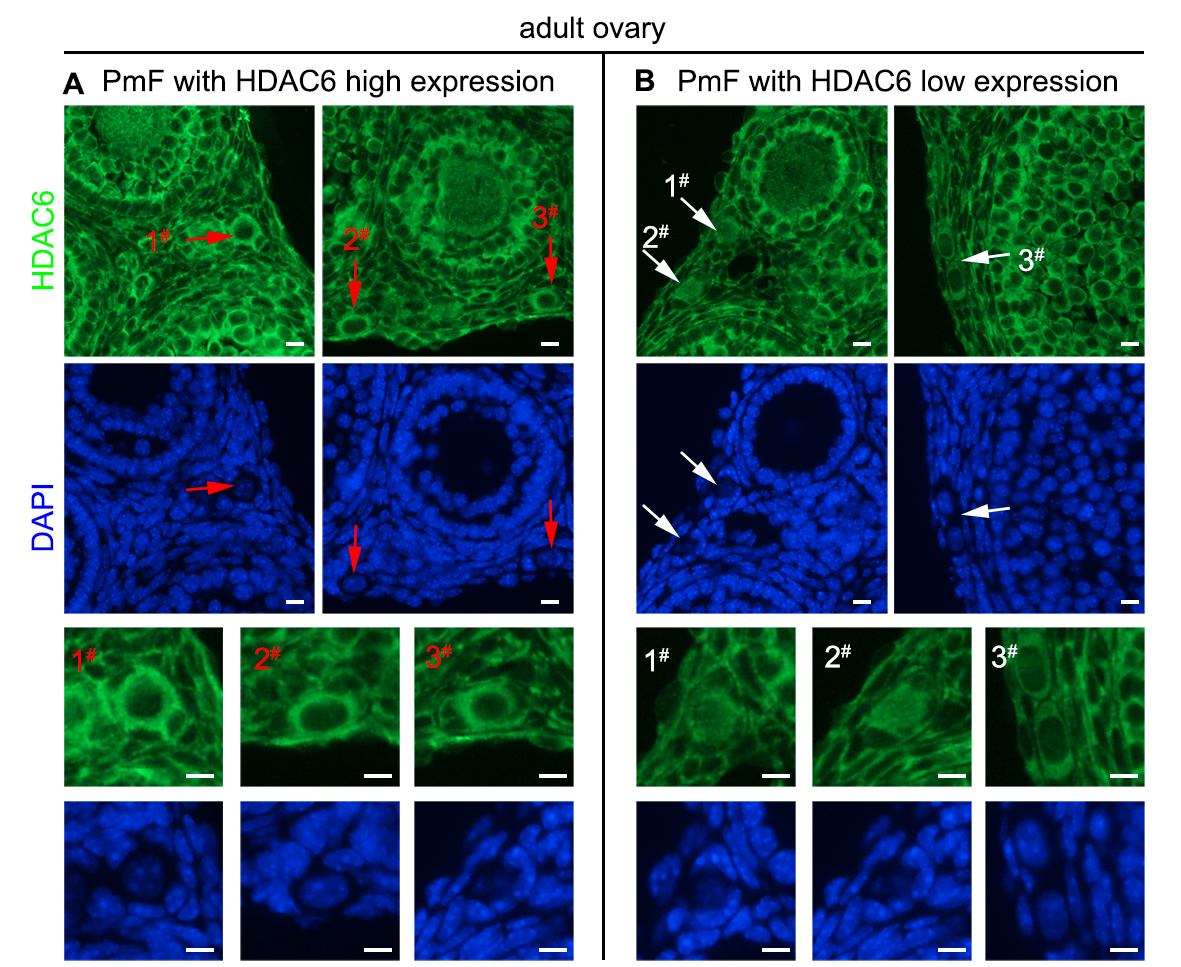

Supplement: Supplementary file 3 — Figure S1 [file 41419_2021_3842_MOESM3_ESM.tif]

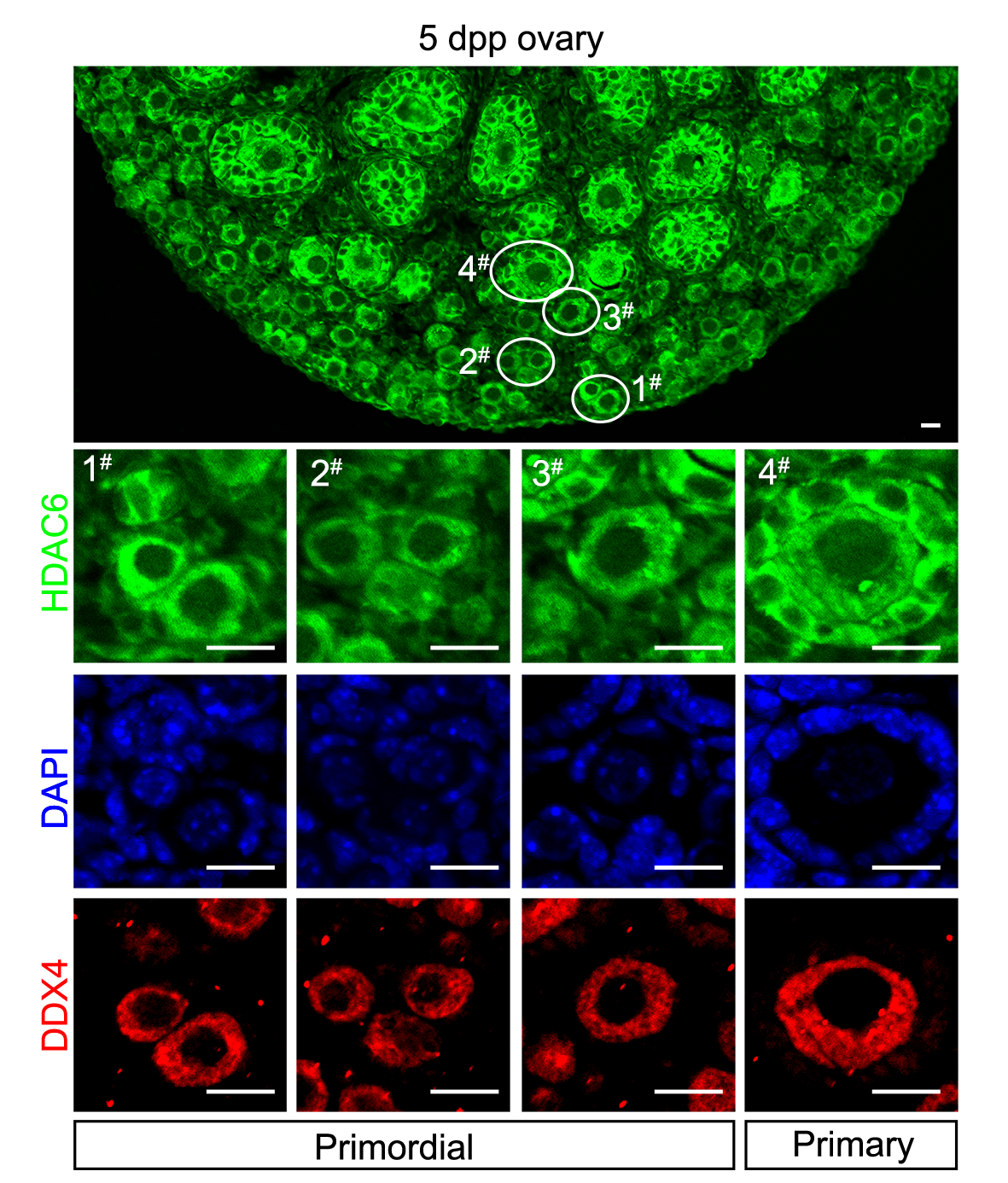

Supplement: Supplementary file 4 — Figure S2 [file 41419_2021_3842_MOESM4_ESM.tif]

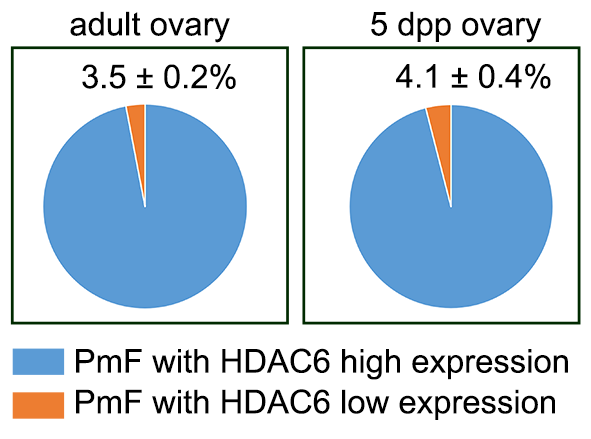

Supplement: Supplementary file 5 — Figure S3 [file 41419_2021_3842_MOESM5_ESM.tif]

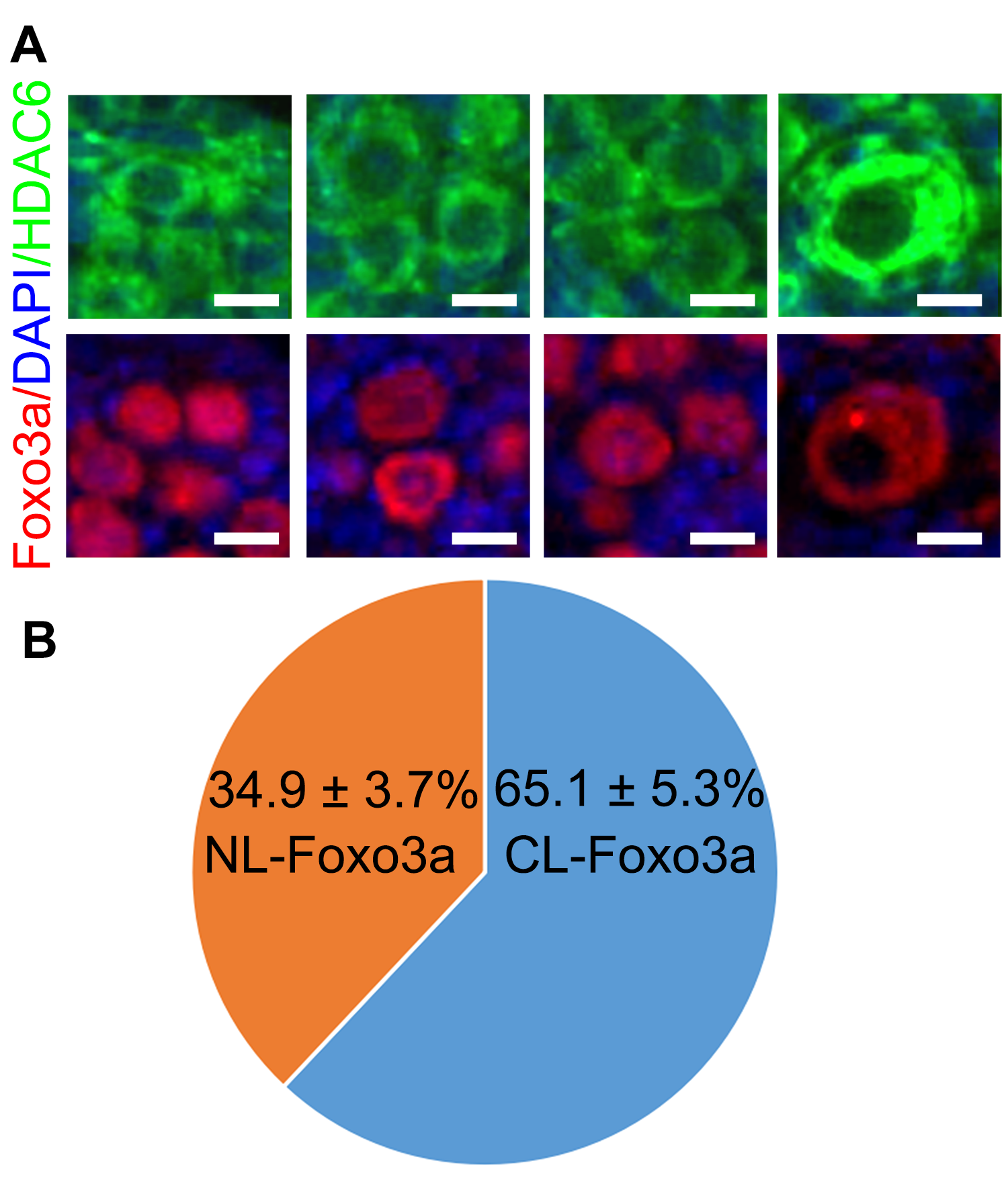

Supplement: Supplementary file 6 — Figure S4 [file 41419_2021_3842_MOESM6_ESM.tif]

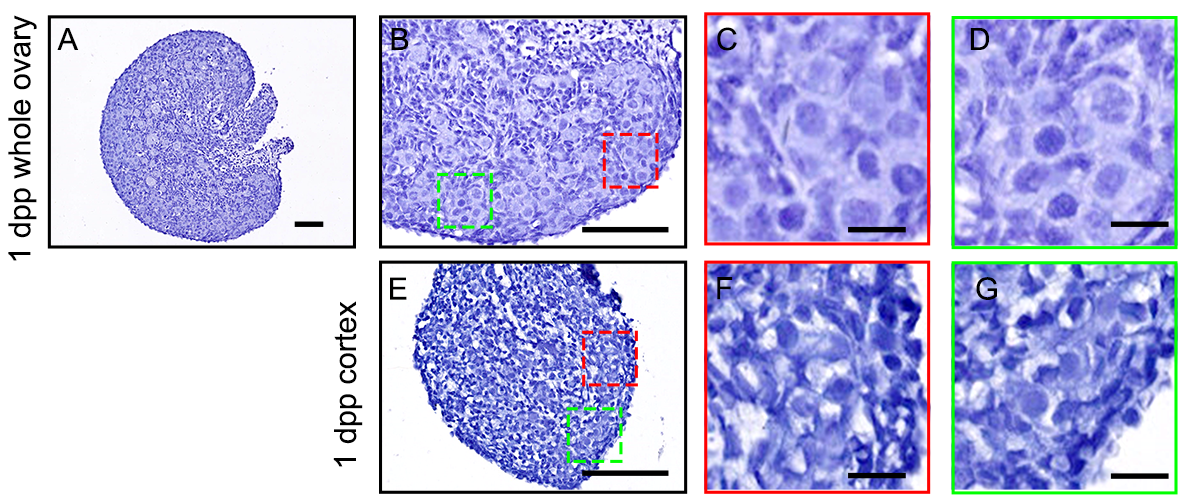

Supplement: Supplementary file 7 — Figure S5 [file 41419_2021_3842_MOESM7_ESM.tif]

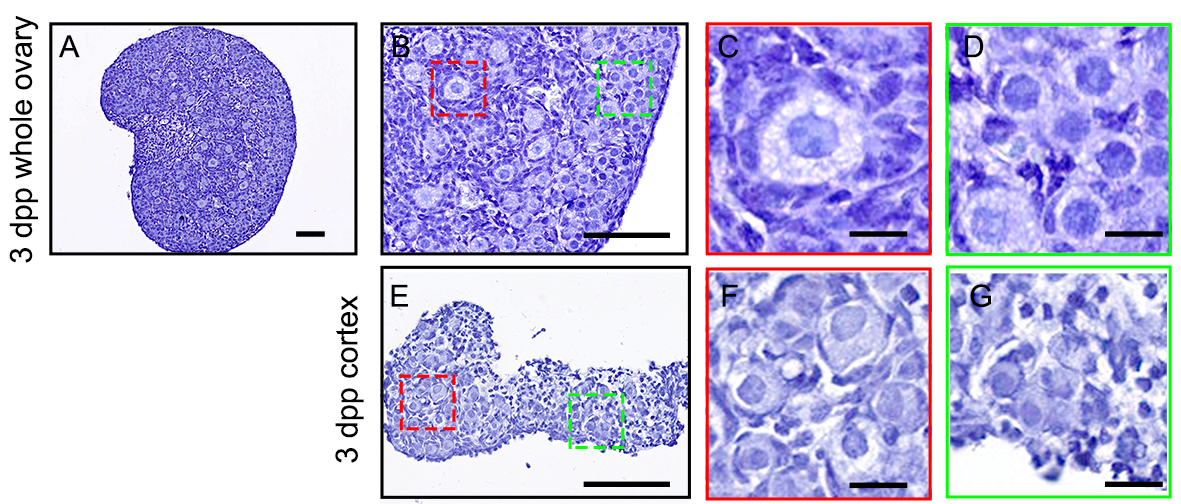

Supplement: Supplementary file 8 — Figure S6 [file 41419_2021_3842_MOESM8_ESM.tif]

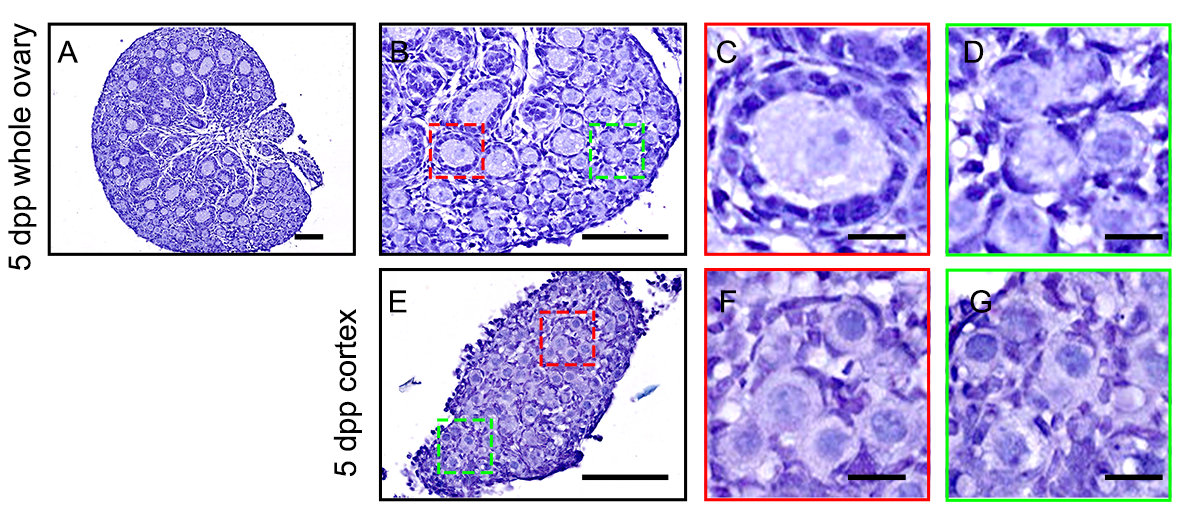

Supplement: Supplementary file 9 — Figure S7 [file 41419_2021_3842_MOESM9_ESM.tif]

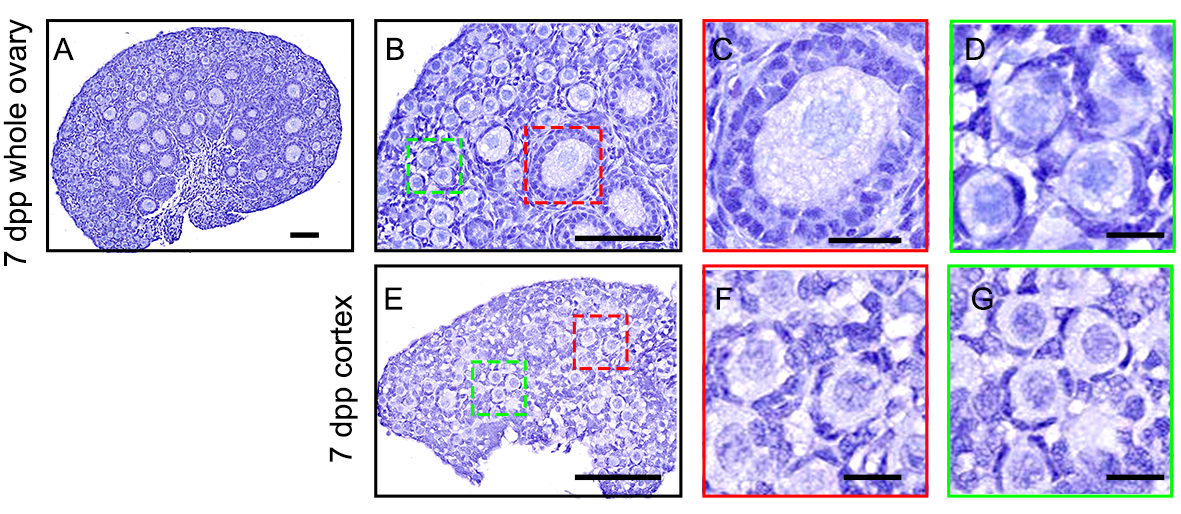

Supplement: Supplementary file 10 — Figure S8 [file 41419_2021_3842_MOESM10_ESM.tif]

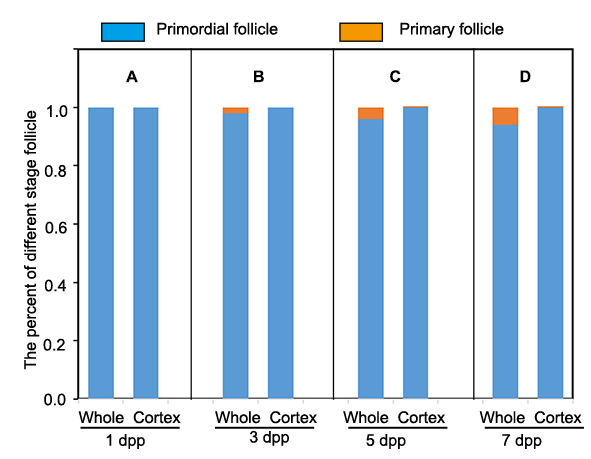

Supplement: Supplementary file 11 — Figure S9 [file 41419_2021_3842_MOESM11_ESM.tif]

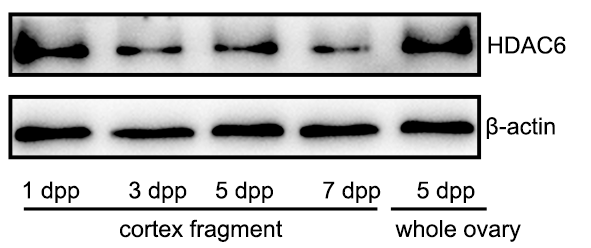

Supplement: Supplementary file 12 — Figure S10 [file 41419_2021_3842_MOESM12_ESM.tif]

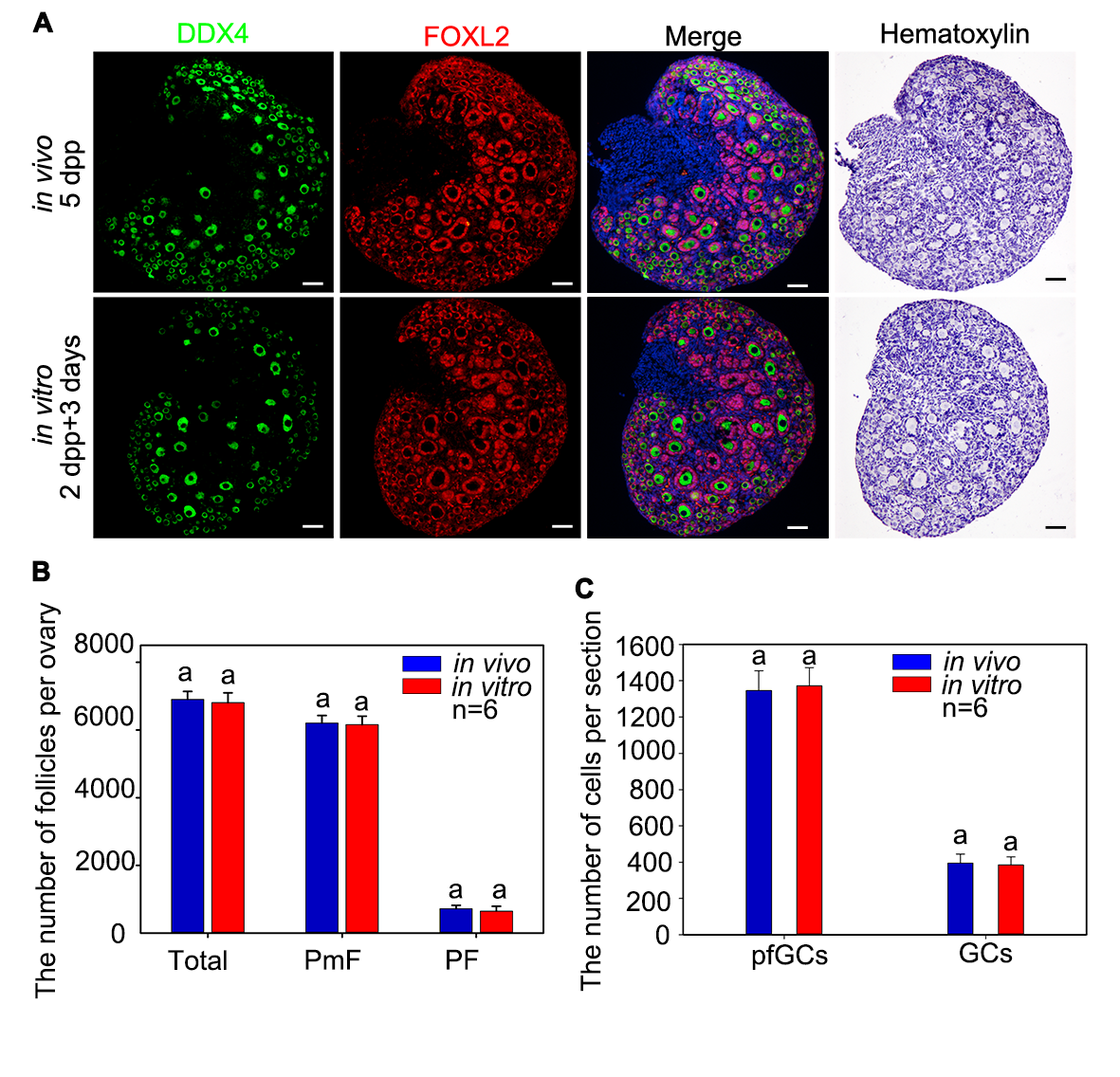

Supplement: Supplementary file 13 — Figure S11 [file 41419_2021_3842_MOESM13_ESM.tif]

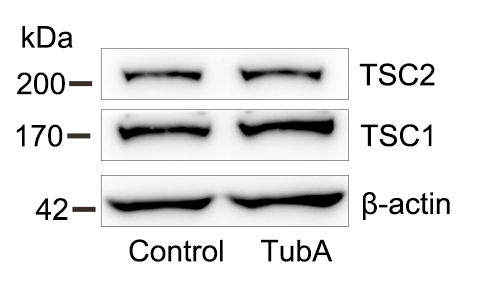

Supplement: Supplementary file 14 — Figure S12 [file 41419_2021_3842_MOESM14_ESM.tif]
